# Higher Sensitivity Provided by the Combination of Two Lateral Flow Immunoassay Tests for the Detection of COVID-19 Immunoglobulins

**DOI:** 10.3389/fcimb.2020.00479

**Published:** 2020-10-21

**Authors:** Ziad Daoud, Jesse McLeod, David L. Stockman

**Affiliations:** ^1^Department of Clinical Microbiology and Infection Prevention, Michigan Health Clinics and Public Health Institute of Science, Epidemiology, and Research, Saginaw, MI, United States; ^2^Faculty of Medicine and Medical Sciences, University of Balamand and Clinical Microbiology Division, Saint George Hospital-University Medical Center (UMC), Beirut, Lebanon

**Keywords:** SARS-CoV-2, IgM, IgG, sensitivity, specificity, serology

## Abstract

SARS-Cov-2 was identified in Wuhan, China in December 2019. The World Health Organization (WHO) declared it a pandemic in March of 2020. COVID-19 has now been reported on every continent. In the United States, the total number of confirmed reported cases of COVID-19 has exceeded 1.8 million with the total death exceeding 100,000 people. The most common investigational diagnostics of this disease are RT-PCR and serology testing. The objective of this work was to validate two commercial kits for the detection of IgM and IgG using lateral flow immunoassay tests and to study the effect of the combination of both serology kits for better detection of immunoglobulins. A total of 195 patients presenting with respiratory symptoms suggestive of infection with SARS-Cov-2 were subject to serology and molecular testing. Two lateral flow immunochromatographic assay kits were used: the Healgen Scientific for SARS-CoV-2 IgM/IgG and the Raybiotech for SARS-CoV-2 IgM/IgG. Sensitivity and specificity of each kit alone and in combination were determined and compared. The limit of detection, inter and intra test variations, as well interfering substances and cross reactivity were also studied for both kits. The results show sensitivities for IgM detection varying between 58.9 and 66.2% for the kits alone and 87.7% of the combination of both kits. IgG detection was not significantly affected by this combination. Both kits manifested high specificities (99.2–100%). Both kits showed high clinical performance in terms of cross reactivity and interfering substances. Our results suggest using combinatory testing for the serology of COVID-19 after a full evaluation study, assessing all the parameters affecting their clinical performance before deciding on this combination.

## Introduction

Severe acute respiratory syndrome coronavirus 2 (SARS-CoV-2) is the virus responsible for the coronavirus disease of 2019 (COVID-19). This virus was first identified in Wuhan, China in December 2019, it has since been declared a pandemic by the World Health Organization (WHO) in March of 2020 (Coronavirus disease (COVID-19) pandemic- 2020 https://www.who.int/emergencies/diseases/novel-coronavirus-2019. Coronavirus disease 2019 (COVID-19): situation summary (2020). https://www.cdc.gov/coronavirus/2019-ncov/cases-updates/summary.html). The disease caused by this virus, COVID-19, has now been reported on every continent. In the United States, the total number of confirmed reported cases of Covid-19 has exceeded 1.8 million with total death exceeding 100,000 people. (https://www.worldometers.info/coronavirus/). The World Health Organization (WHO) has criticized countries that have not prioritized testing. The chief executive of the WHO has highlighted the importance of testing on several occasions (WHO, 2020).

The tests most commonly used now for the diagnosis of COVID-19 are RT-PCR and serology testing. Other techniques such as the detection of the viral antigen are also used. The RT-PCR looks for the virus itself (viral RNA) in the nose, throat, or other areas in the respiratory tract to determine if there is an active infection with SARS-CoV-2 (Liua et al., 2020). A positive PCR test suggests that the person being tested has an active COVID-19 infection.

PCR testing only helps determine whether a person has an active infection at the time of testing (Tahamtan and Ardebili, 2020). Unfortunately, it does not help determine who had an infection in the past. It also does not help determine which people who have been exposed to COVID-19 will develop active infection during the two weeks after exposure. In some people, the virus can only be found by PCR for a few days at the beginning of the infection, so the test might not find the virus if the swab is taken more than a few days after the illness starts. Lateral flow immunoassay (LFIA) testing of SARS-Cov-2 (IgM/IgG) is intended for use as a screening test helping in the identification and diagnosis of human subjects who developed antibodies as a result of SARS-CoV-2 infection or exposure. Any reactive specimen with the IgM-SARS-Cov-2 or IgG-SARS-Cov-2 must be confirmed with alternative testing method(s). It is not yet confirmed if the antibodies produced during this infection will be enough to yield immunity and whether this immunity is long or short. The serology testing should not be used alone to diagnose acute SARS-CoV-2 infection. Antibodies against SARS-CoV-2 can be detected in blood a few days after the infection. The production of IgM typically happens 3–5 days post infection, IgG of course are produced at a later stage; this is known as seroconversion. Negative results do not exclude the possibility of a SARS-CoV-2 infection. For this reason, the combination of both serologic and molecular testing to detect the virus is necessary. Cross reactivity of IgM and/or IgG may occur as a result of a previous exposure to other SARS viruses. The serology testing of SARS-CoV-2 (IgM/IgG) is currently permitted only for use under the Food and Drug Administration's emergency use authorization (EUA) (https://www.cdc.gov/coronavirus/2019-ncov/cases-in-us.html).

As an effective point-of-care tool, paper-based assays offer the advantage of providing supporting results in a timely manner. In addition, these tests are not expensive and allow for faster treatment decisions (Yager et al., 2006). Paper assays have been used in many diagnostic areas. In view of their complexity, many differences can occur between different kits from different manufacturers.

On April 16, 2020, the White House released a document: “Opening Up America Again Guidelines.” This constitutes a road map based on three phases for reopening the society in the States. The Blueprint document released by the White House demonstrates “how the use of two antibody tests rather than one dramatically improves the predictive value of a testing program, particularly in low prevalence environments.” By definition, higher positive predictive values (PPV) are mostly associated with people who contracted the disease and responded with the production of antibodies. Higher negative predictive values (NPV), on the other hand, commonly suggest that one does not have the disease and did not produce the corresponding antibodies (Blueprint for testing plans and rapid response programs- partnering with states to put America back to work https://www.whitehouse.gov/wp-content/uploads/2020/04/Testing-Blueprint.pdf).

In this paper, our intention was to validate two commercial kits for the detection of IgM and IgG using lateral flow immunoassay tests. Based on our results, we propose to combine two serology kits for better detection of immunoglobulins.

## Materials and Methods

Patients and patient sampling: 195 patients presenting with respiratory symptoms suggestive of an infection with SARS-Cov-2 were subject to serology and molecular testing. For serology testing, venous blood was withdrawn by phlebotomy. Blood was collected in EDTA tubes and separated by centrifugation at 2,500 g for 5 min, and plasma was obtained. For molecular testing, a nasopharyngeal swab was obtained from the same patient. RT-PCR experiments were not performed in our lab, they were sent to a reference lab and results were communicated within 24 h.

Lateral flow immunoassay testing: Two different rapid tests were used for the detection IgM and IgG in human plasma. Both test devices utilize lateral flow immunoassay technology that is used for the qualitative, differential detection of both anti-SARS-CoV-2 IgM and IgG antibodies: 1- Healgen Scientific for SARS-CoV-2 IgM/IgG and 2- Raybiotech for SARS-CoV-2 IgM/IgG.

For both kits, the separation of components was performed using capillary force and the specific and rapid binding of an antibody to its antigen. Each cassette consists of a dry medium coated with novel coronavirus N protein and goat anti-chicken IgY antibody (control). Two free colloidal gold-labeled antibodies, mouse anti-human immunoglobulin and chicken IgY, were included in the release pad section. After the addition of plasma, the Ig will bind to coronavirus Ig antibodies if they are present, forming an IgM-IgM complex. The sample and antibodies will then move across the cassette's medium *via* capillary action. If the coronavirus IgM antibody is present in the sample, the IgM-IgM complex will bind to the test line and develop color.

For the Healgen kit, the test cassette was provided in a sealed foil pouch and laid on a flat surface. Using the plastic dropper provided, 5 μl of plasma specimen was transferred into the sample well (S). Immediately 50 μL of sample buffer was added to the buffer well (B) ensuring that the buffer vial tip did not touch the sample, air bubbles were avoided. After, the control line (C) changed from blue to red in color. One additional drop of sample buffer may be added to the buffer well if migration of the sample has not moved across the test window. The results were read in 10 min, no reading was allowed after 15 min. Both IgM and IgG were reported in the following way: 1- Positive for anti-SARS-CoV-2 immunoglobulins when both the control line (C) and the test line (T) were dark or light pink. 2- Negative for anti-SARS-CoV-2 immunoglobulins when the control line (C) was dark pink and the test line (T) did not develop color or had a faint gray band. 3- Invalid: there was no colored control line (C). Image 1 represents the possible lateral flow device results for the IgM and IgG cassette.

For the Raybiotech kit, one test cassette for each immunoglobulin was provided in a sealed foil pouch (One cassette for IgM and another for IgG). For both immunoglobulins, 25 ul of patient plasma were added to a diluent tube that was mixed by gentle inversion. Using the pipette provided with the cassette, 2–3 drops of the prepared diluent/plasma were added to the release pad of the cassette. If there is no movement of the liquid in the first 30 s, an additional drop may be added to the release pad. Plasma from patients with PCR confirmed positive and negative SARS-CoV-2 infection were used as controls. Positive and negative control plasma were frozen at −20°C for up to 3 months in 35 ul aliquots in labeled plastic bullets. For longer storage periods, controls were frozen at −80°C. These were tested with every new kit lot received, after receipt of a new shipment of the same lot, and on daily basis when running tests from patients. Results were read within 10 min, and no reading was done after 15 min. Both IgM and IgG were reported in the following way: 1- Positive for anti-SARS-CoV-2 immunoglobulins: both the control line (C) and the test line (T) were dark or light pink. 2- Negative for anti-SARS-CoV-2 immunoglobulins: the control line (C) was dark pink and the test line (T) did not develop color or had a faint gray band. 3- Invalid: there was no colored control line (C).

When both tests were combined for interpretation, the following rule was implemented for both IgM and IgG: in the case of agreement between both kits (Raybiotech and Healgen), the agreed upon result was reported. In the case of disagreement between both kits (positive for one kit and negative for the other), the result was reported as positive for the immunoglobulin in question.

Performance parameters

1. Sensitivity or PPA (positive coincidence rate) and specificity or NPA (negative coincidence rate) of the separate and combined kits' results were calculated according to the following formulas:
  $$\begin{array}{l} \text{Sensitivity \%}=100\;\times \;\left[\text{True Positive}/\left(\text{True Positive}+\text{False Negative}\right)\right]\\ \text{Specificity \%}=100\;\times \;\left[\text{True Negative}/\left(\text{True Negative}+\text{False Positive}\right)\right] \end{array}$$
  The total agreement with PCR results was calculated in percent of the total coincidence between serology and molecular tests.
2. Accuracy and limit of detection (LoD) for IgM and IgG: three serial dilutions from three different patients' plasma were prepared separately. The dilution interval from one dilution to another was the double leading to decreasing concentrations by half. Detection of IgM tests were performed on all the dilutions and results were recorded.
3. Intra-assay validation (intra-assay repeatability): intra-assay validation shows the reproducibility between the tubes within one testing time. Data resulting from intra-assay validation helps ensure that samples run in different tubes of the same experiment will give comparable results. Eight plasma samples were run in five repetitions each.
4. Cross-reactivity: we evaluated the cross-reactivity of the SARS-Cov-2 detection rapid test using plasma samples from patients with documented antibodies against the below listed pathogens. Human Coronavirus 229 E, Human Coronavirus NL63 (alpha coronavirus, Human Coronavirus, Respiratory Syncytial Virus, Human Coronavirus NL63+RSV, Human Coronavirus 229+RSV, Human Metapneumovirus (hMPV, Parainfluenza virus (1,3,), Influenza A, Influenza B, Hepatitis C Virus, Hepatitis B Virus, Hemophilus influenzae, Chlamydia pneumoniae, HPV, HIV.
5. Class specificity: we evaluated the potential for: (a) human IgM (0.4 mg/ml of human IgM purified immunoglobulin- Biorad) to cross react and produce false positive results for IgG: using five patients' plasma with IgG negative. Each sample was tested in duplicate, (b) human IgG (8 mg/ml of natural human IgG - Biorad) to cross react and produce false positive results for IgM: using five patients' plasma with IgM negative. Each sample was tested in duplicate, and (c) human IgM 0.4 mg/ml and human IgG 8 mg/ml to compete and produce false negative results for IgM or IgG using five patients' plasma (IgM positive IgG positive). Each sample was tested in duplicate.
6. Potential interfering substances: we prepared low titer SARS-CoV-2 antibody positive serum samples, and SARS-CoV-2 antibody negative serum samples. Then we spiked aliquots of both preparations with one of the below substances to approximate the indicated concentrations and tested triplicates. Hemoglobin (10–20 mg/mL), Bilirubin Conjugated <1 mg/mL, Bilirubin Unconjugated <1 mg/mL, Ciprofloxacin 200 mg/L, Cefotaxime 500 mg/L, Meropenem 200 mg/L, Imipenem 200 mg/L, Amikacin 10 mg/L, and Amphotericin B 200 mg/L.
7. Statistical methods: for the comparison of sensitivity and specificity, the Fisher-Exact test was used for the sample population analysis using a two sided *p*. Significance was determined according to the value of *p*.

## Results

The results of the detection of IgM and IgG of 195 tests performed by LFIA using the Raybiotech kit and or the Healgen kit are shown in Tables 1, 2. Concerning IgM detection, Tables 3–5 show that the sensitivity of Raybiotech alone was 58.9%, and Healgen alone was 66.2% (*p*-value for IgM RayBio vs. IgM Healgen = 0.3969). When the rule about disagreement was implemented (refer to Material and Methods), the sensitivity of the combined kits reached 87.7% (*p*-value for IgM RayBio vs. combination of tests = 0.0001 and *p*-value for IgM Healgen vs. combination of tests = 0.0003). These results show a clear significance and beneficial added value for the combination of both kits in detecting IgM.

**Table 1:** Detection of IgM by Raybiotech alone, Healgen alone, Raybiotech + Healgen combined.

**Patient** **plasma**	**RT-PCR**	**IgM Raybio**	**IgM Healgen**	**Comparison RT-PCR/ IgM** **Raybio**	**Comparison RT-PCR/ IgM** **Healgen**	**Comparison RT-PCR-IgM** **Raybio+Healgen**
CP1	Neg	Neg	Neg	A	A	A
CP2	Pos	Pos	Pos	A	A	A
CP3	Pos	Neg	Pos	F-	A	AC
CP4	Neg	Neg	Neg	A	A	A
CP5	Neg	Neg	Neg	A	A	A
CP6	Neg	Neg	Neg	A	A	A
CP7	Pos	Neg	Pos	F-	A	AC
CP8	Pos	Neg	Neg	F-	F-	F-
CP9	Pos	Neg	Neg	F-	F-	F-
CP10	Neg	Neg	Neg	A	A	A
CP11	Neg	Neg	Neg	A	A	A
CP12	Neg	Neg	Neg	A	A	A
CP13	Pos	Neg	Neg	F-	F-	F-
CP14	Neg	Neg	Neg	A	A	A
CP15	Neg	Neg	Neg	A	A	A
CP16	Pos	Pos	Neg	A	F-	AC
CP17	Pos	Pos	Pos	A	A	A
CP18	Pos	Pos	Pos	A	A	A
CP19	Neg	Neg	Neg	A	A	A
CP20	Pos	Pos	Pos	A	A	A
CP21	Neg	Pos	Neg	F+	A	F+-
CP22	Neg	Neg	Neg	A	A	A
CP23	Neg	Neg	Neg	A	A	A
CP24	Pos	Pos	Neg	A	F-	AC
CP25	Neg	Neg	Neg	A	A	A
CP26	Pos	Pos	Pos	A	A	A
CP27	Pos	Neg	Pos	F-	A	AC
CP28	Pos	Pos	Neg	A	F-	AC
CP29	Pos	Neg	Pos	F-	A	AC
CP30	Pos	Neg	Pos	F-	A	AC
CP31	Pos	Pos	Neg	A	F-	AC
CP32	Pos	Pos	Pos	A	A	A
CP33	Pos	Pos	Pos	A	A	A
CP34	Pos	Pos	Neg	A	F-	AC
CP35	Pos	Neg	Neg	F-	F-	F-
CP36	Pos	Neg	Pos	F-	A	AC
CP37	Pos	Pos	Pos	A	A	A
CP38	Pos	Pos	Neg	A	F-	AC
CP39	Pos	Pos	Neg	A	F-	AC
CP40	Pos	Pos	Pos	A	A	A
CP41	Pos	Neg	Neg	F-	F-	F-
CP42	Pos	Neg	Neg	F-	F-	F-
CP43	Pos	Pos	Pos	A	A	A
CP44	Neg	Neg	Neg	A	A	A
CP45	Pos	Pos	Pos	A	A	A
CP46	Pos	Neg	Pos	F-	A	AC
CP47	Pos	Pos	Pos	A	A	A
CP48	Pos	Pos	Pos	A	A	A
CP49	Pos	Neg	Pos	F-	A	AC
CP50	Pos	Neg	Neg	F-	F-	F-
CP51	Neg	Neg	Neg	A	A	A
CP52	Neg	Neg	Neg	A	A	A
CP53	Neg	Neg	Neg	A	A	A
CP54	Neg	Neg	Neg	A	A	A
CP55	Neg	Neg	Neg	A	A	A
CP56	Neg	Neg	Neg	A	A	A
CP57	Neg	Neg	Neg	A	A	A
CP58	Neg	Neg	Neg	A	A	A
CP59	Neg	Neg	Neg	A	A	A
CP60	Neg	Neg	Neg	A	A	A
CP61	Pos	Pos	Pos	A	A	A
CP62	Pos	Pos	Pos	A	A	A
CP63	Neg	Neg	Neg	A	A	A
CP64	Pos	Pos	Pos	A	A	A
CP65	Neg	Neg	Neg	A	A	A
CP66	Neg	Neg	Neg	A	A	A
CP67	Pos	Pos	Pos	A	A	A
CP68	Neg	Neg	Neg	A	A	A
CP69	Neg	Neg	Neg	A	A	A
CP70	Neg	Neg	Neg	A	A	A
CP71	Pos	Pos	Pos	A	A	A
CP72	Neg	Neg	Neg	A	A	A
CP73	Neg	Neg	Neg	A	A	A
CP74	Neg	Neg	Neg	A	A	A
CP75	Neg	Neg	Neg	A	A	A
CP76	Pos	Pos	Neg	A	F-	AC
CP77	Pos	Pos	Neg	A	F-	AC
CP78	Pos	Neg	Pos	F-	A	AC
CP79	Neg	Neg	Neg	A	A	A
CP80	Neg	Neg	Neg	A	A	A
CP81	Pos	Neg	Pos	F-	A	AC
CP82	Neg	Neg	Neg	A	A	A
CP83	Pos	Pos	Pos	A	A	A
CP84	Neg	Neg	Neg	A	A	A
CP85	Pos	Pos	Pos	A	A	A
CP86	Pos	Pos	Pos	A	A	A
CP87	Pos	Pos	Pos	A	A	A
CP88	Pos	Pos	Pos	A	A	A
CP89	Pos	Pos	Pos	A	A	A
CP90	Pos	Pos	Pos	A	A	A
CP91	Pos	Neg	Neg	F-	F-	A
CP92	Pos	Neg	Neg	F-	F-	A
CP93	Pos	Neg	Pos	F-	A	AC
CP94	Pos	Neg	Neg	F-	F-	A
CP95	Pos	Pos	Neg	A	F-	AC
CP96	Pos	Neg	Neg	F-	F-	F-
CP97	Pos	Neg	Neg	F-	F-	F-
CP98	Pos	Pos	Pos	A	A	A
CP99	Pos	Neg	Pos	F-	A	AC
CP100	Pos	Neg	Pos	F-	A	AC
CP101	Neg	Neg	Neg	A	A	A
CP102	Neg	Neg	Neg	A	A	A
CP103	Neg	Neg	Neg	A	A	A
CP104	Neg	Neg	Neg	A	A	A
CP105	Neg	Neg	Neg	A	A	A
CP106	Neg	Neg	Neg	A	A	A
CP107	Neg	Neg	Neg	A	A	A
CP108	Neg	Neg	Neg	A	A	A
CP109	Pos	Pos	Pos	A	A	A
CP110	Pos	Pos	Pos	A	A	A
CP111	Pos	Pos	Neg	A	F-	AC
CP112	Pos	Neg	Pos	F-	A	AC
CP113	Pos	Neg	Pos	F-	A	AC
CP114	Pos	Pos	Pos	A	A	A
CP115	Pos	Pos	Pos	A	A	A
CP116	Pos	Neg	Pos	F-	A	AC
CP117	Pos	Neg	Neg	F-	F-	A
CP118	Pos	Pos	Pos	A	A	A
CP119	Pos	Pos	Pos	A	A	A
CP120	Pos	Neg	Pos	F-	A	AC
CP121	Neg	Neg	Neg	A	A	A
CP122	Neg	Neg	Neg	A	A	A
CP123	Neg	Neg	Neg	A	A	A
CP124	Neg	Neg	Neg	A	A	A
CP125	Neg	Neg	Neg	A	A	A
CP126	Neg	Neg	Neg	A	A	A
CP127	Neg	Neg	Neg	A	A	A
CP128	Neg	Neg	Neg	A	A	A
CP129	Neg	Neg	Neg	A	A	A
CP130	Neg	Neg	Neg	A	A	A
CP131	Neg	Neg	Neg	A	A	A
CP132	Neg	Neg	Neg	A	A	A
CP133	Neg	Neg	Neg	A	A	A
CP134	Neg	Neg	Neg	A	A	A
CP135	Neg	Neg	Neg	A	A	A
CP136	Neg	Neg	Neg	A	A	A
CP137	Neg	Neg	Neg	A	A	A
CP138	Neg	Neg	Neg	A	A	A
CP139	Neg	Neg	Neg	A	A	A
CP140	Neg	Neg	Neg	A	A	A
CP141	Neg	Neg	Neg	A	A	A
CP142	Neg	Neg	Neg	A	A	A
CP143	Neg	Neg	Neg	A	A	A
CP144	Neg	Neg	Neg	A	A	A
CP145	Neg	Neg	Neg	A	A	A
CP146	Neg	Neg	Neg	A	A	A
CP147	Neg	Neg	Neg	A	A	A
CP148	Neg	Neg	Neg	A	A	A
CP149	Neg	Neg	Neg	A	A	A
CP150	Neg	Neg	Neg	A	A	A
CP151	Neg	Neg	Neg	A	A	A
CP152	Neg	Neg	Neg	A	A	A
CP153	Neg	Neg	Neg	A	A	A
CP154	Neg	Neg	Neg	A	A	A
CP155	Neg	Neg	Neg	A	A	A
CP156	Neg	Neg	Neg	A	A	A
CP157	Neg	Neg	Neg	A	A	A
CP158	Neg	Neg	Neg	A	A	A
CP159	Neg	Neg	Neg	A	A	A
CP160	Neg	Neg	Neg	A	A	A
CP161	Neg	Neg	Neg	A	A	A
CP162	Neg	Neg	Neg	A	A	A
CP163	Neg	Neg	Neg	A	A	A
CP164	Neg	Neg	Neg	A	A	A
CP165	Neg	Neg	Neg	A	A	A
CP166	Neg	Neg	Neg	A	A	A
CP167	Neg	Neg	Neg	A	A	A
CP168	Neg	Neg	Neg	A	A	A
CP169	Neg	Neg	Neg	A	A	A
CP170	Neg	Neg	Neg	A	A	A
CP171	Neg	Neg	Neg	A	A	A
CP172	Neg	Neg	Neg	A	A	A
CP173	Neg	Neg	Neg	A	A	A
CP174	Neg	Neg	Neg	A	A	A
CP175	Neg	Neg	Neg	A	A	A
CP176	Neg	Neg	Neg	A	A	A
CP177	Neg	Neg	Neg	A	A	A
CP178	Neg	Neg	Neg	A	A	A
CP179	Neg	Neg	Neg	A	A	A
CP180	Neg	Neg	Neg	A	A	A
CP181	Neg	Neg	Neg	A	A	A
CP182	Neg	Neg	Neg	A	A	A
CP183	Neg	Neg	Neg	A	A	A
CP184	Neg	Neg	Neg	A	A	A
CP185	Neg	Neg	Neg	A	A	A
CP186	Neg	Neg	Neg	A	A	A
CP187	Neg	Neg	Neg	A	A	A
CP188	Neg	Neg	Neg	A	A	A
CP189	Neg	Neg	Neg	A	A	A
CP190	Neg	Neg	Neg	A	A	A
CP191	Neg	Neg	Neg	A	A	A
CP192	Neg	Neg	Neg	A	A	A
CP193	Neg	Neg	Neg	A	A	A
CP194	Neg	Neg	Neg	A	A	A
CP195	Neg	Neg	Neg	A	A	A

*Neg, Negative; Pos, Positive; F-, False negative; F+, False positive; A, Agreement with PCR result; AC, Agreement of combined testing*.

**Table 2:** Detection of IgG by Raybiotech alone, Healgen alone, Raybiotech + Healgen combined.

**Patient** **plasma**	**RT-PCR**	**IgG RayBio**	**IgG Healgen**	**Comparison RT-PCR/IgG** **Raybio**	**Comparison RT-PCR/ IgG** **Healgen**	**Comparison RT-PCR/ IgG** **Raybio+Healgen**
CP1	Neg	Neg	Neg	A	A	A
CP2	Pos	Pos	Pos	A	A	A
CP3	Pos	Pos	Pos	A	A	A
CP4	Neg	Neg	Neg	A	A	A
CP5	Neg	Neg	Neg	A	A	A
CP6	Neg	Neg	Neg	A	A	A
CP7	Pos	Neg	Neg	F-	F-	F-
CP8	Pos	Neg	Neg	F-	F-	F-
CP9	Pos	Neg	Neg	F-	F-	F-
CP10	Neg	Neg	Neg	A	A	A
CP11	Neg	Neg	Neg	A	A	A
CP12	Neg	Neg	Neg	A	A	A
CP13	Pos	Neg	Neg	F-	F-	F-
CP14	Neg	Neg	Neg	A	A	A
CP15	Neg	Neg	Neg	A	A	A
CP16	Pos	Pos	Pos	A	A	A
CP17	Pos	Pos	Pos	A	A	A
CP18	Pos	Neg	Neg	F-	F-	F-
CP19	Neg	Neg	Neg	A	A	A
CP20	Pos	Pos	Pos	A	A	A
CP21	Neg	Neg	Neg	A	A	A
CP22	Neg	Neg	Neg	A	A	A
CP23	Neg	Neg	Neg	A	A	A
CP24	Pos	Neg	Neg	F-	F-	F-
CP25	Neg	Neg	Neg	A	A	A
CP26	Pos	Pos	Pos	A	A	A
CP27	Pos	Neg	Neg	F-	F-	F-
CP28	Pos	Neg	Neg	F-	F-	F-
CP29	Pos	Pos	Pos	A	A	A
CP30	Pos	Pos	Pos	A	A	A
CP31	Pos	Neg	Neg	F-	F-	F-
CP32	Pos	Pos	Pos	A	A	A
CP33	Pos	Pos	Pos	A	A	A
CP34	Pos	Neg	Neg	F-	F-	F-
CP35	Pos	Neg	Neg	F-	F-	F-
CP36	Pos	Neg	Pos	F-	A	AC
CP37	Pos	Pos	Pos	A	A	A
CP38	Pos	Neg	Neg	F-	F-	F-
CP39	Pos	Neg	Neg	F-	F-	F-
CP40	Pos	Pos	Pos	A	A	A
CP41	Pos	Neg	Neg	F-	F-	F-
CP42	Pos	Neg	Neg	F-	F-	F-
CP43	Pos	Pos	Pos	A	A	A
CP44	Neg	Neg	Neg	A	A	A
CP45	Pos	Pos	Pos	A	A	A
CP46	Pos	Pos	Pos	A	A	A
CP47	Pos	Neg	Neg	F-	F-	F-
CP48	Pos	Pos	Pos	A	A	A
CP49	Pos	Pos	Pos	A	A	A
CP50	Pos	Neg	Neg	F-	F-	F-
CP51	Neg	Neg	Neg	A	A	A
CP52	Neg	Neg	Neg	A	A	A
CP53	Neg	Neg	Neg	A	A	A
CP54	Neg	Neg	Neg	A	A	A
CP55	Neg	Neg	Neg	A	A	A
CP56	Neg	Neg	Neg	A	A	A
CP57	Neg	Neg	Neg	A	A	A
CP58	Neg	Neg	Neg	A	A	A
CP59	Neg	Neg	Neg	A	A	A
CP60	Neg	Neg	Neg	A	A	A
CP61	Pos	Pos	Pos	A	A	A
CP62	Pos	Pos	Pos	A	A	A
CP63	Neg	Neg	Neg	A	A	A
CP64	Pos	Pos	Pos	A	A	A
CP65	Neg	Neg	Neg	A	A	A
CP66	Neg	Neg	Neg	A	A	A
CP67	Pos	Pos	Pos	A	A	A
CP68	Neg	Neg	Neg	A	A	A
CP69	Neg	Neg	Neg	A	A	A
CP70	Neg	Neg	Neg	A	A	A
CP71	Pos	Neg	Pos	F-	A	AC
CP72	Neg	Neg	Neg	A	A	A
CP73	Neg	Neg	Neg	A	A	A
CP74	Neg	Neg	Neg	A	A	A
CP75	Neg	Neg	Neg	A	A	A
CP76	Pos	Pos	Pos	A	A	A
CP77	Pos	Pos	Pos	A	A	A
CP78	Pos	Pos	Pos	A	A	A
CP79	Neg	Neg	Neg	A	A	A
CP80	Neg	Neg	Neg	A	A	A
CP81	Pos	Pos	Pos	A	A	A
CP82	Neg	Neg	Neg	A	A	A
CP83	Pos	Pos	Pos	A	A	A
CP84	Neg	Neg	Neg	A	A	A
CP85	Pos	Pos	Pos	A	A	A
CP86	Pos	Neg	Pos	F-	A	AC
CP87	Pos	Pos	Pos	A	A	A
CP88	Pos	Pos	Pos	A	A	A
CP89	Pos	Pos	Pos	A	A	A
CP90	Pos	Pos	Pos	A	A	A
CP91	Pos	Pos	Pos	A	A	A
CP92	Pos	Pos	Pos	A	A	A
CP93	Pos	Pos	Pos	A	A	A
CP94	Pos	Pos	Pos	A	A	A
CP95	Pos	Neg	Neg	F-	F-	F-
CP96	Pos	Pos	Pos	A	A	A
CP97	Pos	Pos	Pos	A	A	A
CP98	Pos	Pos	Pos	A	A	A
CP99	Pos	Pos	Pos	A	A	A
CP100	Pos	Pos	Pos	A	A	A
CP101	Neg	Neg	Neg	A	A	A
CP102	Neg	Neg	Neg	A	A	A
CP103	Neg	Neg	Neg	A	A	A
CP104	Neg	Neg	Neg	A	A	A
CP105	Neg	Neg	Neg	A	A	A
CP106	Neg	Neg	Neg	A	A	A
CP107	Neg	Neg	Neg	A	A	A
CP108	Neg	Neg	Neg	A	A	A
CP109	Pos	Pos	Pos	A	A	A
CP110	Pos	Pos	Pos	A	A	A
CP111	Pos	Pos	Pos	A	A	A
CP112	Pos	Pos	Pos	A	A	A
CP113	Pos	Pos	Pos	A	A	A
CP114	Pos	Pos	Pos	A	A	A
CP115	Pos	Pos	Pos	A	A	A
CP116	Pos	Pos	Pos	A	A	A
CP117	Pos	Neg	Neg	F-	F-	F-
CP118	Pos	Pos	Pos	A	A	A
CP119	Pos	Pos	Pos	A	A	A
CP120	Pos	Pos	Pos	A	A	A
CP121	Neg	Neg	Neg	A	A	A
CP122	Neg	Neg	Neg	A	A	A
CP123	Neg	Neg	Neg	A	A	A
CP124	Neg	Neg	Neg	A	A	A
CP125	Neg	Neg	Neg	A	A	A
CP126	Neg	Neg	Neg	A	A	A
CP127	Neg	Neg	Neg	A	A	A
CP128	Neg	Neg	Neg	A	A	A
CP129	Neg	Neg	Neg	A	A	A
CP130	Neg	Neg	Neg	A	A	A
CP131	Neg	Neg	Neg	A	A	A
CP132	Neg	Neg	Neg	A	A	A
CP133	Neg	Neg	Neg	A	A	A
CP134	Neg	Neg	Neg	A	A	A
CP135	Neg	Neg	Neg	A	A	A
CP136	Neg	Neg	Neg	A	A	A
CP137	Neg	Neg	Neg	A	A	A
CP138	Neg	Neg	Neg	A	A	A
CP139	Neg	Neg	Neg	A	A	A
CP140	Neg	Neg	Neg	A	A	A
CP141	Neg	Neg	Neg	A	A	A
CP142	Neg	Neg	Neg	A	A	A
CP143	Neg	Neg	Neg	A	A	A
CP144	Neg	Neg	Neg	A	A	A
CP145	Neg	Neg	Neg	A	A	A
CP146	Neg	Neg	Neg	A	A	A
CP147	Neg	Neg	Neg	A	A	A
CP148	Neg	Neg	Neg	A	A	A
CP149	Neg	Neg	Neg	A	A	A
CP150	Neg	Neg	Neg	A	A	A
CP151	Neg	Neg	Neg	A	A	A
CP152	Neg	Neg	Neg	A	A	A
CP153	Neg	Neg	Neg	A	A	A
CP154	Neg	Neg	Neg	A	A	A
CP155	Neg	Neg	Neg	A	A	A
CP156	Neg	Neg	Neg	A	A	A
CP157	Neg	Neg	Neg	A	A	A
CP158	Neg	Neg	Neg	A	A	A
CP159	Neg	Neg	Neg	A	A	A
CP160	Neg	Neg	Neg	A	A	A
CP161	Neg	Neg	Neg	A	A	A
CP162	Neg	Neg	Neg	A	A	A
CP163	Neg	Neg	Neg	A	A	A
CP164	Neg	Neg	Neg	A	A	A
CP165	Neg	Neg	Neg	A	A	A
CP166	Neg	Neg	Neg	A	A	A
CP167	Neg	Neg	Neg	A	A	A
CP168	Neg	Neg	Neg	A	A	A
CP169	Neg	Neg	Neg	A	A	A
CP170	Neg	Neg	Neg	A	A	A
CP171	Neg	Neg	Neg	A	A	A
CP172	Neg	Neg	Neg	A	A	A
CP173	Neg	Neg	Neg	A	A	A
CP174	Neg	Neg	Neg	A	A	A
CP175	Neg	Neg	Neg	A	A	A
CP176	Neg	Neg	Neg	A	A	A
CP177	Neg	Neg	Neg	A	A	A
CP178	Neg	Neg	Neg	A	A	A
CP179	Neg	Neg	Neg	A	A	A
CP180	Neg	Neg	Neg	A	A	A
CP181	Neg	Neg	Neg	A	A	A
CP182	Neg	Neg	Neg	A	A	A
CP183	Neg	Neg	Neg	A	A	A
CP184	Neg	Neg	Neg	A	A	A
CP185	Neg	Neg	Neg	A	A	A
CP186	Neg	Neg	Neg	A	A	A
CP187	Neg	Neg	Neg	A	A	A
CP188	Neg	Neg	Neg	A	A	A
CP189	Neg	Neg	Neg	A	A	A
CP190	Neg	Neg	Neg	A	A	A
CP191	Neg	Neg	Neg	A	A	A
CP192	Neg	Neg	Neg	A	A	A
CP193	Neg	Neg	Neg	A	A	A
CP194	Neg	Neg	Neg	A	A	A
CP195	Neg	Neg	Neg	A	A	A

*Neg, Negative; Pos, Positive; F-, False negative; F+, False positive; A, Agreement with PCR result; AC, Agreement of combined testing*.

**Table 3:** Sensitivity, specificity, and agreement of Raybiotech LFIA IgM with RT-PCR results.

		**LFIA IgM (Raybio)**		**Total**
		**+**	**-**	
RT-PCR	+	43	30	73
	-	1	121	122
	Total	44	151	195
	Sensitivity	Specificity	Agreement	
	58.9%	99.2%	84.1%	

**Table 4:** Sensitivity, specificity, and agreement of Healgen LFIA IgM with RT-PCR results.

		**LFIA IgM (Healgen)**		**Total**
		**+**	**-**	
RT-PCR	+	49	25	74
	-	0	121	121
	Total	49	146	195
	Sensitivity	Specificity	Agreement	
	66.2%	100.0%	87.7%	

**Table 5:** Sensitivity, specificity, and agreement of combined Raybiotech+Healgen LFIA IgM with RT-PCR results.

		**LFIA IgM combined**		**Total**
		**+**	**-**	
RT-PCR	+	64	9	73
	-	1	121	122
	Total	65	130	195
	Sensitivity	Specificity	Agreement	
	87.7%	99.2%	94.9%	

Concerning IgG, as shown in Tables 6–8, it was noted that the sensitivity of Raybiotech alone was 68.9%, and Healgen alone was 74.0% (*p*-value for IgG RayBio vs. IgG Healgen = 0.5847). When the rule about disagreement was implemented (refer to Material and Methods), the sensitivity of the combined kits was 74.0% (*p*-value of IgG Raybio vs. combination of both tests = 0.5847). This result does not suggest an added value for the combination of both kits in detecting IgG. The very low number of false positive results (only one patient) resulted in a high specificity varying between 99.2 and 100% for both kits.

**Table 6:** Sensitivity, specificity, and agreement of Raybiotech LFIA IgG with RT-PCR results.

		**LFIA IgG (Raybio)**		**Total**
		**+**	**-**	
RT-PCR	+	51	23	74
	-	0	121	121
	Total	51	144	195
		Sensitivity	Specificity	Agreement
		68.9%	100.0%	88.7%

**Table 7:** Sensitivity, specificity, and agreement of Healgen LFIA IgG with RT-PCR results.

		**LFIA IgG (Healgen)**		**Total**
		**+**	**-**	
RT-PCR	+	54	19	73
	-	0	122	122
	Total	54	141	195
		Sensitivity	Specificity	Agreement
		74.0%	100.0%	90.3%

**Table 8:** Sensitivity, specificity, and agreement of combined Raybiotech+Healgen LFIA IgG with RT-PCR results.

		**LFIA IgG combined**		**Total**
		**+**	**-**	
RT-PCR	+	54	19	73
	-	0	122	122
	Total	54	141	195
		Sensitivity	Specificity	Agreement
		74.0%	100.0%	90.3%

The results of limit of detection show a better detection of IgG at higher dilutions of the sample in both kits than for IgM (Tables 9, 10).

**Table 9:** Limit of detection for IgM and IgG by Healgen kit.

	**Dilutions of the plasma of patients CP2, CP17, and CP64**						
**CP2**	**D0**	**D1**	**D2**	**D3**	**D4**	**D5**	**D6**
Concentration	1	D0/2	D0/4	D0/8	D0/16	D0/32	D0/64
Band control	+	+	+	+	+	+	-
Band IgM	+	+	+	-	-	-	-
Band IgG	+	+	+	+	+	+	-
**CP17**	**D0**	**D1**	**D2**	**D3**	**D4**	**D5**	**D6**
Concentration	1	D0/2	D0/4	D0/8	D0/16	D0/32	D0/64
Band control	+	+	+	+	+	+	-
Band IgM	+	+	+	-	-	-	-
Band IgG	+	+	+	+	+	+	-
**CP64**	**D0**	**D1**	**D2**	**D3**	**D4**	**D5**	**D6**
Concentration	1	D0/2	D0/4	D0/8	D0/16	D0/32	D0/64
Band control	+	+	+	+	+	+	-
Band IgM	+	+	+	-	-	-	-
Band IgG	+	+	+	+	+	+	-

**Table 10:** Limit of detection for IgM and IgG by Raybiotech kit.

	**Dilutions of the plasma of patients CP2, CP17, and CP64**						
**CP2**	**D0**	**D1**	**D2**	**D3**	**D4**	**D5**	**D6**
Concentration	1	D0/2	D0/4	D0/8	D0/16	D0/32	D0/64
Band control	+	+	+	+	+	+	+
Band IgM	+	+	+	-	-	-	-
Band IgG	+	+	+	+	+	+	-
**CP17**	**D0**	**D1**	**D2**	**D3**	**D4**	**D5**	**D6**
Concentration	1	D0/2	D0/4	D0/8	D0/16	D0/32	D0/64
Band control	+	+	+	+	+	+	+
Band IgM	+	+	+	+	+	+	-
Band IgG	+	+	+	+	+	+	+
**CP64**	**D0**	**D1**	**D2**	**D3**	**D4**	**D5**	**D6**
Concentration	1	D0/2	D0/4	D0/8	D0/16	D0/32	D0/64
Band control	+	+	+	+	+	+	+
Band IgM	+	+	+	+	+	-	-
Band IgG	+	+	+	+	+	+	+

For both tested kits, no cross-reactivity of the SARS-CoV-2 detection rapid tests using plasma samples from patients with documented antibodies against the below listed pathogens was seen: Human Coronavirus 229 E, Human Coronavirus NL63 (alpha coronavirus, Human Coronavirus, Respiratory Syncytial Virus, Human Coronavirus NL63+RSV, Human Coronavirus 229+RSV, Human Metapneumovirus (hMPV, Parainfluenza virus (1,3,), Influenza A, Influenza B, Hepatitis C Virus, Hepatitis B Virus, Hemophilus influenzae, Chlamydia pneumoniae, HPV, HIV.

No class specificity interference with both kits was found using human IgM and human IgG. No false positive or false negative results were recorded.

None of the tested substances (Hemoglobin, Bilirubin Conjugated, Bilirubin Unconjugated, Ciprofloxacin, Cefotaxime, Meropenem, Imipenem, Amikacin, and Amphotericin) were found to interfere with the ability of IgM and IgG detection in both kits. No changes in results were recorded for the inter- and intra-assay tests. All replicates of the same samples in the same experiment as well as the same samples in different experiments yielded the same results.

## Discussion

The pandemic caused by SARS-CoV-2 has been ongoing since the end of 2019. Unfortunately, in the absence of a successful vaccine and a standard efficient treatment, this virus remains invincible in many ways. Lockdown of societies and prevention measures only hope to contain the transmission of the disease until further notice. In this context, massive testing remains a good strategy to identify carriers of the virus. In view of the shortage of material and resources, RT-PCR tests should be part of the algorithm of testing and not the only test. Serology tests investigating the development of IgG and IgM in patients who have been potentially infected is another good addition to the algorithm. Unfortunately, many parameters including the onset of the disease, incubation period, symptomatology, and immunity status of the patient put some limitations on these serological tests regarding their sensitivity and specificity (Haveri et al., 2020). A negative IgM, negative IgG test can be interpreted as no evidence of an increase in human IgM and IgG production against SARS-CoV-2. This negative or non-reactive result might indicate a state of no infection or an incubation period of the virus. In the case of suspicious exposure, the patient should be advised to repeat the test in 7–10 days (Pal et al., 2020). A negative result can also be due to a delayed immune response by the patient. A test showing positive IgM and negative or non-reactive IgG can indicate an early stage of a SARS-CoV-2 infection. The absence of IgG indicates that the patient had not developed acquired immunity yet. A positive IgM and IgG result suggests either an active or an early recovery stage of the infection with SARS-CoV-2. A negative IgM and positive IgG generally indicates a late stage or a past infection with SARS-CoV-2. This suggests that an acquired immunity has developed to the virus.

The detection of immunoglobulins (M and G) is based on immunochromatography where the separation of components in a mixture is accomplished based on the capillary force and the highly specific antigen-antibody binding. IgM antibodies to SARS-CoV-2 generally become positive (detectable in serum) between day 5 and 7 following infection but may occur later. This is the same for IgG antibodies to SARS-CoV-2 that generally become detectable 10–14 days following infection (Huang et al., 2020).

In this work, we have shown that the combination of two lateral flow immunochromatographic tests increase the sensitivity of the assay in detecting IgM, however, this was not the case for IgG. As well as any other testing, the sensitivity and specificity of paper-based assays determines their performance (Tan et al., 1999). Sensitivity assesses and measures the ability of the test to correctly detect the target-substrate. In this context, it is very important to determine the limit of detection (LoD) which can directly affect positively or negatively the sensitivity of the test in question (Wang et al., 2013; Farka et al., 2017). The LoD corresponds to the lowest concentration associated with a positive detectable signal. Several parameters can influence the LoD, such as the affinity of the antigen and antibody, as well as the physical properties of these molecules. In addition, the paper substrate properties, number, printed detector molecules, immunoprobe stability, readout method, and competition with free target molecules are all parameters that can influence the quality of the lateral flow immunochromatography test.

On the other hand, the specificity of a technique relates to the probability of yielding a false positive result. Similar to sensitivity, it is defined by many parameters and factors including the cross-reactivity and nonspecific binding to the immunoprobe. On the other hand, fluidic properties of the paper strip as well as sample preparation are important parameters affecting paper assay performance (Hristov et al., 2019).

It is expected that different manufacturers will produce different products with different performances. When two tests are used for the same sample, there is the possibility of agreement or disagreement on the result. While agreement between tests is considered to “dramatically improve the predictive value of a testing program, particularly in low prevalence environments” (Blueprint for testing plans and rapid response programs- partnering with states to put America back to work https://www.whitehouse.gov/wp-content/uploads/2020/04/Testing-Blueprint.pdf), the disagreement can also be used to dramatically improve the agreement of LFIA with RT-PCT tests, as shown in our paper. This effect has been significantly high with IgM (Tables 3–8) and was insignificant with IgG. Our results suggest that in view of the differences in material and production, and in view of the high specificity of immunoglobulin detection (yielding low levels of false positive results), it is to our advantage to use more than one kit for the detection of immunoglobulins and consider the positive result where the disagreement occurs. The reason why this is true for IgM and not for IgG might be due to the high specificity, smaller size, and higher number of IgGs as compared to IgMs. In any case, the combination of more than one kit should be only advised if this is confirmed by method evaluation and validation.

Regarding the evaluation of LFIA for SARS-CoV-2 detection by Raybiotech and Healgen, our results have shown particularly good performance in terms of the limit of detection, interfering substances, and inter and intra assay variation. These parameters are extremely important in the evaluation process, not only for their direct significance, but also for their influence on the sensitivity and specificity of the tests.

In conclusion, our results are in agreement with others suggesting the use of combinatory testing for the serology of COVID-19 and suggest a full evaluation study including all the parameters affecting their clinical performance before deciding on this combination.

## Data Availability Statement

All datasets generated for this study are included in the article/supplementary material.

## Ethics Statement

The studies involving human participants were reviewed and approved by Institution Review Board waived- Michigan Health Clinics IRB approval waived- Only plasma samples previously collected from the patients were used. Written informed consent to participate in this study was provided by the participants' legal guardian/next of kin.

## Author Contributions

ZD designed the study, participated in the experiments, data analysis, and manuscript writing. JM participated in the experiments and data analysis. DS participated in the study design, data analysis, and manuscript writing. All authors contributed to the article and approved the submitted version.

## Conflict of Interest

The authors declare that the research was conducted in the absence of any commercial or financial relationships that could be construed as a potential conflict of interest.
